# Optimisation of a key cross-coupling reaction towards the synthesis of a promising antileishmanial compound

**DOI:** 10.1016/j.tetlet.2019.03.068

**Published:** 2019-05-02

**Authors:** Raul F. Velasco, César Guerrero, Gloria Fra, Alejandra Moure, Juan Miguel-Siles, Maria Teresa Quesada-Campos, Jose Ramon Ruiz-Gomez, Ian H. Gilbert, Michael G. Thomas, Timothy J. Miles

**Affiliations:** a GalChimia S.A., Cebreiro s/n, 15823, O Pino, A Coruña, Spain; b Global Health R&D, GlaxoSmithKline, Calle Severo Ochoa, 2, 28760, Tres Cantos, Madrid, Spain; c Drug Discovery Unit, Wellcome Centre for Anti-Infectives Research, Division of Biological Chemistry and Drug Discovery, School of Life Sciences, University of Dundee, Sir James Black Centre, Dundee DD1 5EH, UK

**Keywords:** Buchwald-Hartwig coupling, Protecting group strategy, Visceral leishmaniasis, 2-(Trimethylsilyl)ethoxymethyl (SEM)

## Abstract

During the course of a research program aimed at identifying novel antileishmanial compounds, a multi-gram synthesis of *N*-(*trans*-4-((4-methoxy-3-((*R*)-3-methylmorpholino)-1*H*-pyrazolo[3,4-*d*]pyrimidin-6-yl)amino)cyclohexyl)-2-methylpropane-1-sulfonamide ((***R***)**-1**) was required. This letter describes optimisation of the reaction conditions and protecting group strategy for a key Buchwald-Hartwig coupling, delivering the required quantities of (***R***)**-1**, as well as further compounds in the series.

## Highlights

• Buchwald-Hartwig coupling of 3-bromo-1*H*-pyrazolo[3,4-*d*]pyrimidine with 3-methylmorpholine.
• Optimisation led to RuPhos, KHMDS and SEM as the optimal protecting group.
• These conditions delivered 9 g of (***R***)**-1** in high purity plus close analogues.

## Graphical abstract

**Figure:**
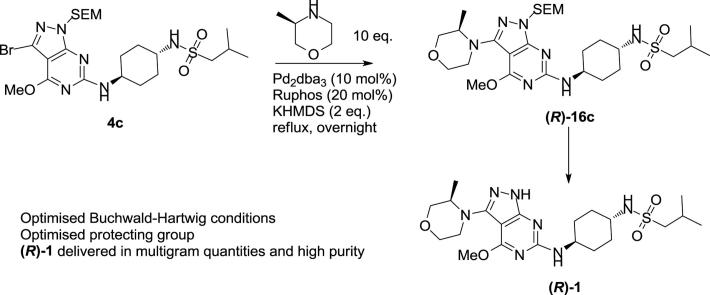

## Introduction

**Scheme 1:**
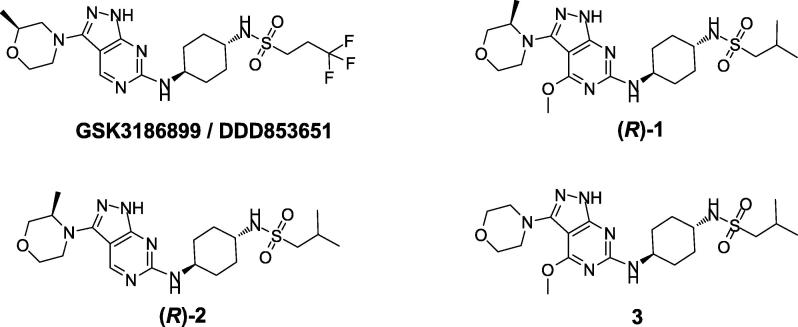
A selection of *N*-1-(1*H*-pyrazolo[3,4-*d*]pyrimidin-6-yl)cyclohexyl-1,4-*trans*-diamine compounds of interest.

During the course of a research program aimed at identifying novel antileishmanial compounds, we discovered a series of N1-(1*H*-pyrazolo[3,4-*d*]pyrimidin-6-yl)cyclohexyl-1,4-*trans*-diamine compounds that led to **GSK3186899/DDD853651** being selected as a pre-clinical development candidate for the treatment of visceral leishmaniasis (Scheme 1) [1], [2]. During the lead optimisation process, the chemistry team became interested in compound (***R***)**-1** due to the orientation of a 4-methoxypyrimidyl substituent alongside a 3-methylmorpholine in the 3-position of the pyrazole ring. Whilst these groups could be introduced individually in a relatively straightforward manner (*e.g.* 3-methylmorpholine with no methoxy (***R***)**-2** or a methoxy substituent and an unsubstituted morpholine **3**, Scheme 1) incorporating both substituents into a single compound proved to be synthetically challenging.

**Scheme 2:**
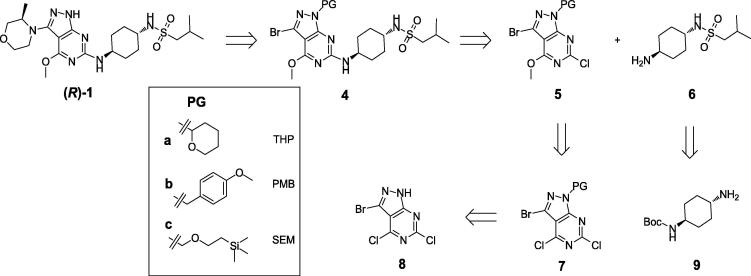
Retrosynthesis of (***R***)**-1**.

The synthetic strategy chosen is highlighted in Scheme 2, starting from 3-bromo-4,6-dichloro-1*H*-pyrazolo[3,4-*d*]pyrimidine **8**; which involved protection of the pyrazole *N*—H followed by sequential displacement of the three halides of **8**. The next step required a Buchwald-Hartwig coupling using 3-methylmorpholine, where there are few examples in the literature [3], particularly when coupled with a sterically hindered partner such as **4**. Herein, we describe the development of the Buchwald-Hartwig coupling which enabled the delivery of multi-gram quantities of (***R***)**-1**, with sufficiently high purity for 7 day rodent toxicology evaluation.

**Scheme 3:**
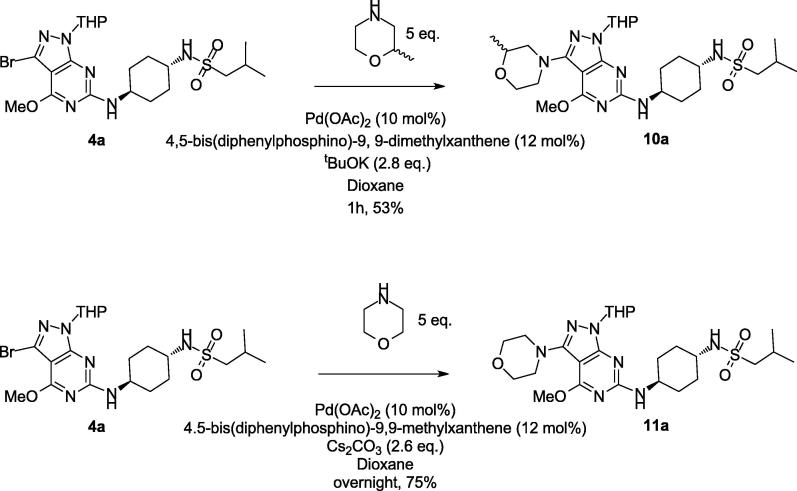
Synthesis of less sterically hindered analogues **10a** and **11a**.

Within the medicinal chemistry program, analogues with less sterically hindered morpholines, such as **10a** and **11a** (Scheme 3), were synthesised *via* standard Buchwald-Hartwig coupling conditions in reasonable yields (48–75%) [2], [4]. However, when these conditions were applied to the more sterically hindered 3-methylmorpholine of interest (working initially on the racemate), only around 20% of product was visible by LCMS in the reaction mixture after heating at reflux overnight, and pure compound could not be isolated from the crude reaction mixture.

**Scheme 4:**
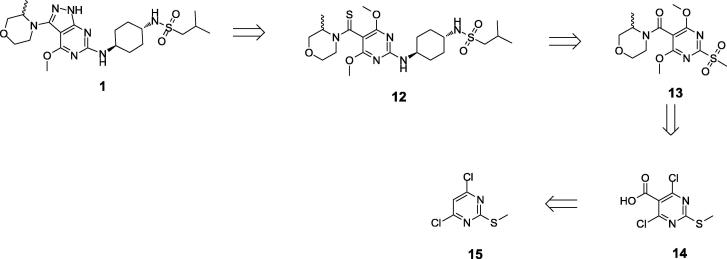
Alternative retrosynthesis of **1**.

Because of this poor yield, an alternative route to the more sterically hindered compounds was investigated. This was based on the alternative retrosynthesis in Scheme 4. The key step would be to construct the pyrazole ring *via* activation and cyclisation of an appropriate amide such as **13**, avoiding the need for the challenging Buchwald-Hartwig coupling. This cyclisation was successfully used for the synthesis of analogues without a 4-methoxypyrimidyl substituent through generation and cyclisation of the thioamide [2]. However in this case, cyclisation did not occur even under forcing conditions, with demethylation of the methoxy groups being observed.

Because the alternative route proved unsuccessful, we returned to the initial Buchwald-Hartwig coupling route (Scheme 2) and undertook an examination of all the components of the reaction: palladium source, ligand, base and solvent. The intent was to identify a set of conditions that could deliver at least 100 mg of final compound.

Table 1 highlights a selection of the conditions tested, demonstrating the range of variables assessed and that the majority of conditions gave poor results. Starting material **4a** was detected by liquid chromatography–mass spectrometry (LCMS) in most cases, alongside two major byproducts: debrominated starting material **17a** and demethylated starting material **18a** (particularly when using sodium *tert*-butoxide or potassium triphosphate as a base). Where product **16a** was detected, the conversion was generally poor (0–23%). The choice of base appeared to be a key factor, as potassium hexamethyldisilazide (KHMDS, a strong non-nucleophilic base) gave significant improvements in conversion (52% by LCMS) with no evidence of remaining **4a** or the byproducts **17a** and **18a**. Cesium fluoride (a non-nucleophilic and less sterically hindered base) gave no conversion to product with only starting material detected, presumably due to its lower base strength compared to KHMDS.

**Table 1:** Effect of alternative conditions on the Buchwald-Hartwig coupling to give **16a**.

**Figure:**
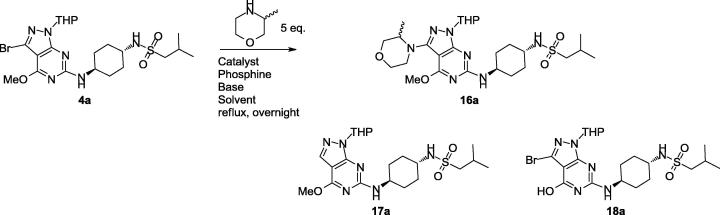

Catalyst	Solvent	Base	Phosphine	**16a**^e^	**4a**^e^	**17a**^e^	**18a**^e^
Pd_2_dba_3_	Dioxane	Cs_2_CO_3_	Xantphos	5	16	50	–
			Xantphos^a^	7	45	30	–
			RuPhos	4	52	27	–
		KHMDS	Ruphos	**54**	–	–	–
	DME	Cs_2_CO_3_	Xantphos	6	23	49	–
			RuPhos	19	21	40	–
		*^t^*BuONa	BINAP	0	–	–	53
			DPEPhos	0	–	–	45
	Toluene	Cs_2_CO_3_	RuPhos	9	60	25	–

Pd(OAc)_2_	Dioxane	Cs_2_CO_3_	Xantphos^b^	22	–	51	–
			Xantphos^a^	17	43	–	–
		*^t^*BuOK	Xantphos^a^	21	24	33	–
		KHMDS	Ruphos^c^	**52**	–	–	–
	DME	*^t^*BuONa	SPhos	0	50	5	25
			tBuXPhos	0	34	–	25
			2-(dicyclohexylphosphino)biphenyl	0	20	–	56
		K_3_PO_4_	SPhos	0	68	17	8
			Brettphos	0	58	30	10
		Cs_2_CO_3_	Ruphos	0	16	40	–
	Toluene	Cs_2_CO_3_	Xantphos	0	23	18	–
			RuPhos	0	15	9	–
		*^t^*BuONa	BINAP^d^	0	28	–	39

Reactions were carried out on 50 mg scale using **4a**, 5 mol% catalyst, 3.5 eq. base, 5 eq. racemic 3-methylmorpholine and 10 mol% ligand.

a 10 mol% catalyst, 2.8 eq. base, 10 eq. racemic 3-methylmorpholine and 12 mol% ligand.

b 10 mol% catalyst, 2.8 eq. base, 5 eq. racemic 3-methylmorpholine and 12 mol% ligand.

c 5 mol% catalyst, 1.0 eq. base, 5 eq. racemic 3-methylmorpholine and 10 mol% ligand.

d 10 mol% catalyst, 3.0 eq. base, 5 eq. racemic 3-methylmorpholine and 10 mol% ligand. DME is dimethoxyethane. Crude reaction mixtures were analyzed by LCMS to determine the relative percentages of starting material, product and side-products.

e % by LCMS.

Using the identified KHMDS conditions on larger scale (1 g) delivered 190 mg (18% isolated yield) of **16a**, which was subsequently tetrahydropyran (THP) deprotected to give **1**.

Although these conditions delivered sufficient compound for early profiling, our continued interest in (***R***)**-1** required us to further optimize the challenging cross-coupling for a multi-gram and high-purity synthesis. We therefore undertook further refinement of the different variables of the reaction. In order to increase the reactivity of the palladium source, various palladacycles were investigated [5], [6], based on reports of significant improvements to Buchwald-Hartwig couplings. Unfortunately, in all cases tested, no improvement was observed. Moreover, 2nd Generation Buchwald-Hartwig ligands (*e.g.* JohnPhos or SPhos) [7], 3rd (*e.g.* BrettPhos, tBuXPhos, Xantphos) [7] and 4th generation ligands (*e.g.* Josiphos) [8], [9] also failed to improve product formation. Investigation of other reaction variables including the loading of the palladium catalyst added, the ratio of catalyst to phosphine ligand, reaction temperature (reduced to 80 °C) and solvent (DME, *n*-butanol, toluene, propylene glycol, 1,3-dimethyl-2-imidazolidinone (DMI) or dioxane); all resulted in little improvement. Varying the number of equivalents of 3-methylmorpholine was observed to have some influence on yield, whereby increasing to 10 equivalents increased the isolated yield to 29% when other variables were kept the same.

This examination of reaction conditions gave a broad coverage of the standard conditions used in Buchwald-Hartwig couplings with limited impact on yields; so finally, the effect of the protecting group on the coupling was examined. This proved to have a major impact on the feasibility of the cross-coupling. The original THP protecting group was replaced with either *p*-methoxybenzyl (PMB) or 2-(trimethylsilyl)ethoxymethyl (SEM). Attempts were also made to protect the *N*—H with tosyl, but this proved challenging to introduce and was not further pursued. After a small amount of optimization of the number of equivalents of the various reagents, both PMB and SEM gave a significant increase in the isolated yield of the cross-coupling (*i.e.* PMB [39%] and SEM [53%] compared to THP [18%]) (Table 2).

**Table 2:** Effect of alternative protecting groups to give **1**.

**Figure:**
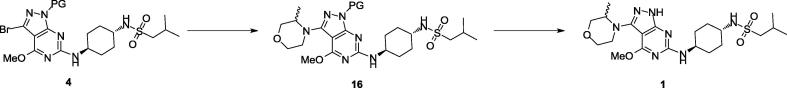

Protecting Group	(a) THP	(b) PMB	(c) SEM
Synthesis of **7** from **8** (protection of the pyrazole *N*—H) (% yield)	76%	17%	98%
Cross-Coupling (**4** to **16**, % yield)	18%	39%	53%
Cross-Coupling Conditions^a^	Pd_2_dba_3_ (7.5 mol%) Ruphos (15 mol%) KHMDS (3 eq.)3-Methylmorpholine (10 eq.)	Pd_2_dba_3_ (5 mol%) Ruphos (10 mol%) KHMDS (2 eq.)3-Methylmorpholine (10 eq.)	Pd_2_dba_3_ (10 mol%) Ruphos (20 mol%) KHMDS (2 eq.)3-Methylmorpholine (10 eq.)
Purification of the Buchwald product	Column chromatography × 2	Column chromatography × 1 Purity 90%	Column chromatography × 1 Purity >99%
Deprotection Conditions	HCl/MeOH	TFA	AcCl/MeOH
Deprotection (% yield)	84%	96%	70%

a Best conditions for each protecting group is shown.

The final step of the synthesis involved removal of the protecting group, the results of which are summarized in Table 2. Standard SEM deprotection using tetra-*^n^*butylammonium fluoride (TBAF) gave poor yields, whereas the use of acetyl chloride in methanol, after optimisation of the reaction conditions, gave a significant improvement. Also, when considering the overall reaction scheme, including introduction and removal of protecting group, yield of cross-coupling and ease of purification, SEM was determined to be the most favourable group on all counts. Therefore, with a set of optimised conditions in hand, the route was scaled up. The coupling was thus carried out on 35 g of **4c** together with (*R*)-3-methylmorpholine delivering 16.91 g of (***R***)**-16c**, an isolated yield of 47%. Subsequent deprotection gave 9.26 g of high purity (***R***)**-1** in an isolated yield of 70% and 100% *ee*, demonstrating that no racemisation occurred during synthesis.

Finally, the coupling conditions developed were applied to a number of different substrates, as highlighted in Table 3. This further highlighted that the conditions could be used to deliver a wide range of compounds to the medicinal chemistry program, and also that the use of SEM as a protecting group (50–64% yield) gave significantly better results than THP (12–18% yield).

**Table 3:** A range of compounds formed utilising the optimised Buchwald-Hartwig coupling conditions.

Compound		PG	Cross-coupling (g), % isolated yield
	(***S***)**-1**	SEM	12.7 g, 50%
	**19**	THP	1 g, 13%
	(***R***)**-20**	SEM	5 g, 64%
	(***S***)**-20**	SEM	5 g, 62%
	**21**	THP	1.4 g, 12%
	**22**	THP	0.75 g, 12%
	**23**	THP	0.83 g, 18%

## Conclusion

In summary, we required a suitable synthetic route to deliver multi-gram quantities of (***R***)**-1**, which involved the Buchwald-Hartwig coupling of a hindered 3-bromo-1*H*-pyrazolo[3,4-*d*]pyrimidine with 3-methylmorpholine. Optimisation of both the reaction conditions and the protecting group led to the use of RuPhos as catalyst, KHMDS as base and SEM as the optimal protecting group. These conditions were then utilized to deliver more than 9 g of (***R***)**-1** in high purity, as well as being applied to the synthesis of further compounds in the series.
