# Supplementary material for: Optimisation of a key cross-coupling reaction towards the synthesis of a promising antileishmanial compound
Source: Tetrahedron Lett. 2019 May 2;60(18):1243–7. doi: 10.1016/j.tetlet.2019.03.068 (PMC6480136; doi:10.1016/j.tetlet.2019.03.068)

**Supplementary Information**

Chemicals and solvents were purchased from the Aldrich Chemical Co., Fluka, Fluorochem, VWR, Acros, Fisher Chemicals, and Alfa Aesar and were used as received. Air- and moisture-sensitive reactions were carried out under an inert atmosphere of argon in oven-dried glassware. Analytical thin-layer chromatography (TLC) was performed on precoated TLC plates (layer 0.20 mm silica gel 60 with fluorescent indicator UV254, from Merck). Developed plates were air-dried and analyzed under a UV lamp (UV254/365 nm). Flash column chromatography was performed using prepacked silica gel cartridges (230−400 mesh, 40−63 μm, from SiliCycle) using a Teledyne ISCO Combiflash Companion, or Combiflash Retrieve. ^1^H NMR and ^13^C NMR spectra were recorded on a Bruker Avance DPX 500 spectrometer (^1^H at 500.1 MHz, ^13^C at 125.8 MHz). Chemical shifts (δ) are expressed in ppm recorded using the residual solvent as the internal reference in all cases. Signal splitting patterns are described as singlet (s), doublet (d), triplet (t), quartet (q), multiplet (m), broad (b), or a combination thereof. Coupling constants (*J*) are quoted to the nearest 0.1 Hz. LC-MS analyses were performed with either an Agilent HPLC 1100 series connected to a Bruker Daltonics MicrOTOF or an Agilent Technologies 1200 series HPLC connected to an Agilent Technologies 6130 quadrupole LC/MS, where both instruments were connected to an Agilent diode array detector. Mobile phase was water/acetonitrile + 0.1% HCOOH, or water/acetonitrile + 0.1% NH3; linear gradient 80:20 to 5:95 over 3.5 min, and then held for 1.5 min; flow rate 0.5 mL min^−1^. All intermediates had a measured purity of >90% as determined using this analytical LC-MS system unless otherwise noted. All final compounds had a measured purity of ≥95% as determined using this analytical LC-MS system (TIC and UV).

**Sample Coupling.**

***N*-(*trans*-4-((4-methoxy-3-(3-methylmorpholino)-1-((2-(trimethylsilyl)ethoxy)methyl)-1*H*-pyrazolo[3,4-*d*]pyrimidin-6-yl)amino)cyclohexyl)-2-methylpropane-1-sulfonamide 16c**

A solution of **4c** (35 g, 59.2 mmol) in 1,4-Dioxane (1000 mL) was purged with argon, (*R*)-3-methylmorpholine (59.8g, 592 mmol) was added and purged, Tris(dibenzylideneacetone)dipalladium(0) (5.42g, 5.92 mmol) was added and purged, dicyclohexyl(2',6'-diisopropoxy-[1,1'-biphenyl]-2-yl)phosphine (5.52g, 11.83 mmol) was added and purged and finally KHMDS in toluene (237 mL, 118 mmol) was added and purged. The mixture was stirred at 110ºC for 2 hours then filtered through celite and partitioned between ethyl acetate (500 mL) and brine (1 L). The phases were separated and the organic phase washed with further brine (1 L). The aqueous layers were combined and extracted with ethyl acetate (1 L). The organic phases were then combined, dried over Na_2_SO_4_, filtered and concentrated. The resulting brown oil was chromatographed on silica gel with cyclohexane/EtOAc (0-30%), giving **16c** as a pale brown solid (16.91g, 28.9 mmol, 47%). MS (ESI): m/z 612.3 [M + H]^+^

**Sample SEM Deprotection**

***N*-(*trans*-4-((4-methoxy-3-((*R*)-3-methylmorpholino)-1*H*-pyrazolo[3,4-*d*]pyrimidin-6-yl)amino)cyclohexyl)-2-methylpropane-1-sulfonamide (*R*)-1**

To **16c**  (16.8g, 27.5 mmol) in methanol (450 mL) at 0ºC was added acetyl chloride (21.4 mL, 302 mmol) dropwise and the resulting yellow solution stirred at room temperature for 3 hours. The reaction was partitioned between dichloromethane (200 mL) and a saturated solution of NaHCO_3_ (100 mL), and the pH adjusted to 7 by slow addition of 2M NaOH. The phases were separated and the aqueous layer extracted with dichloromethane (100 mL). The organic layers were combined, dried over Na_2_SO_4_, filtered and concentrated. The crude product was chromatographed on silica gel with 0-30% cyclohexane/EtOAc:EtOH (3:1) to give **(*R*)-1** as a pale yellow solid (9.26g, 19.23 mmol, 70.0 %). ^1^H NMR (DMSO-*d6*): δ 11.96 (bs, 1H), 6.98 (d, *J* = 7.4 Hz, 1H), 6.90 (d, *J* = 7.4 Hz ,1H), 3.99 – 3.93 (m, 1H), 3.91 (s, 3H), 3.79 – 3.70 (m, 2H), 3.64 – 3.51 (m, 3H), 3.27 – 3.09 (m, 2H), 3.03 (m, 1H), 2.88 (d, *J* = 6.4 Hz, 2H), 2.12 – 2.01 (sept, *J* = 6.6 Hz, 1H), 1.93 – 1.85 (m, 4H), 1.36 – 1.26 (m, 4H), 1.04 – 0.99 (m, 9H); ^13^C NMR (DMSO-*d*6): δ 162.93, 160.46, 160.09, 150.87, 87.58, 70.97, 66.58, 53.78, 51.89, 51.11, 44.34, 33.36, 31.73, 28.84, 24.97, 22.79, 14.43, 12.85; HRMS (ESI): m/z [M + H]^+^ calcd for C21H36N7O4S, 482.2544; found, 482.2523; m/z 482.3 [M + H]^+^; HPLC: 100 %; Enantioselectivity determined using a chiral column: Chiralpak IA, 150 x 4.6 mm 5u, Heptane/EtOH = 65/35 + 0.2% isopropyl amine, 1.0 mL/min, 298K, 254nm. First enantiomer (major) Rt = 8.5 min, second enantiomer (not detected) Rt = 15.5 min. *ee* measured: 100%.


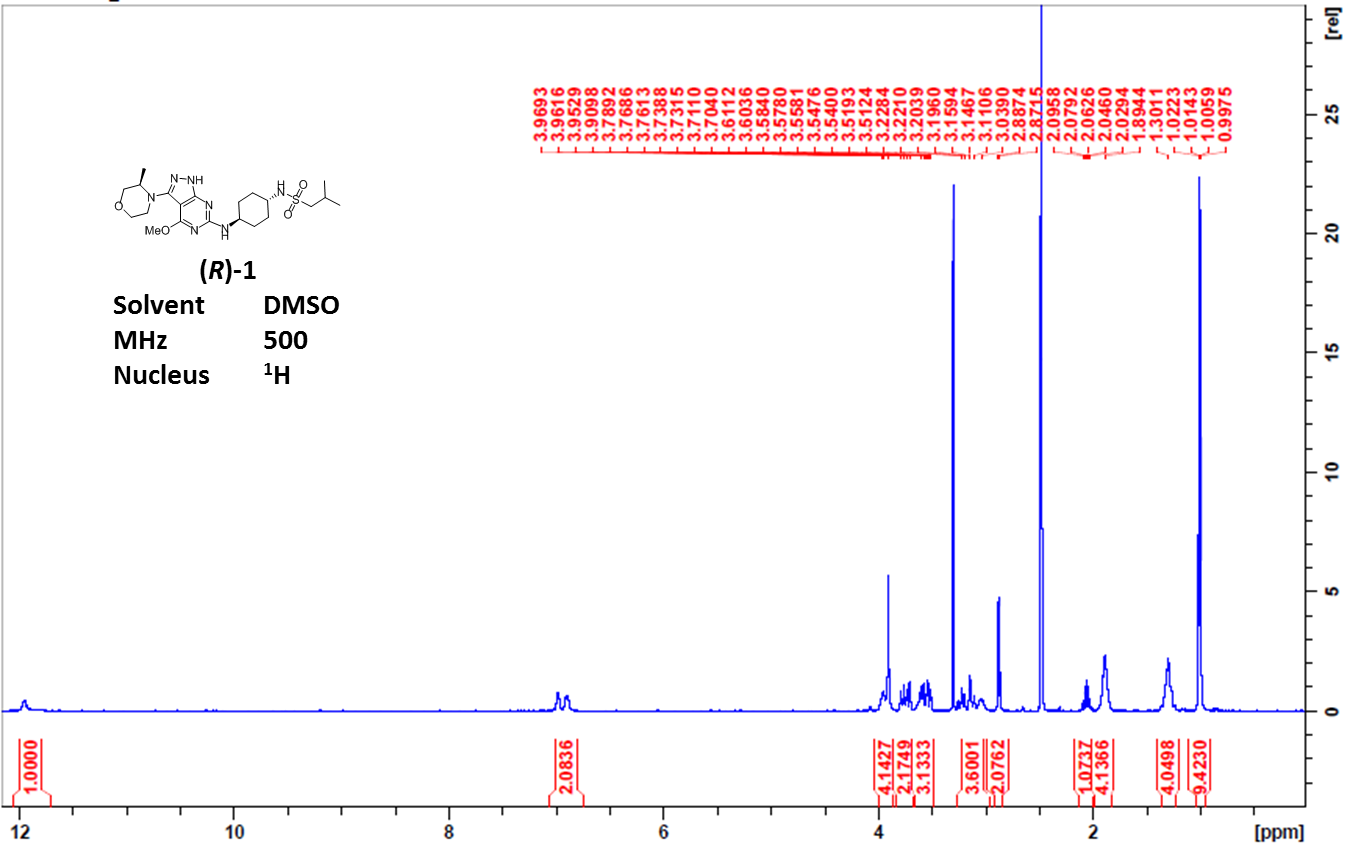


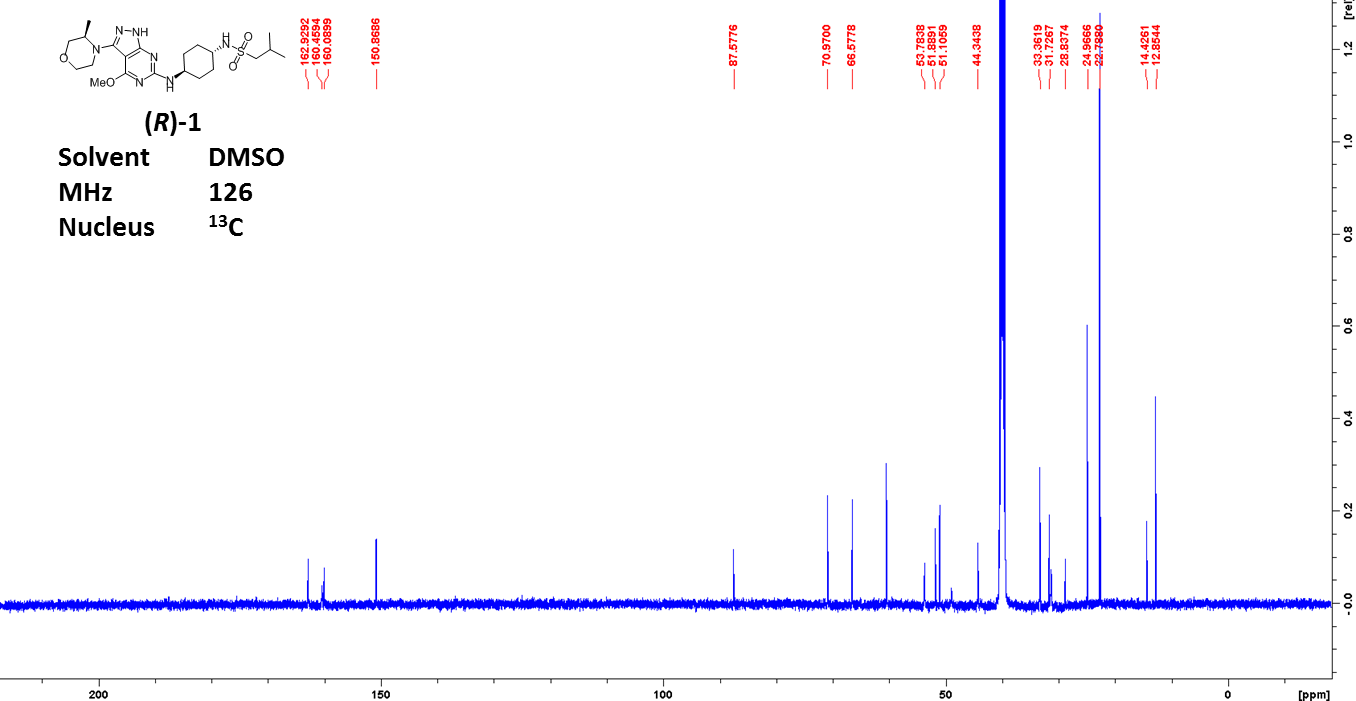


**3-Bromo-4,6-dichloro-1-(tetrahydro-2*H*-pyran-2-yl)-1*H*-pyrazolo[3,4-*d*]pyrimidine 7a**

To a solution of **8** (11.7 g, 43.7 mmol) in THF (218 mL), 3,4-dihydro-2*H*-pyran (11.0 g, 131 mmol) and *p*-toluenesulfonic acid monohydrate (1.5 g, 8.73 mmol) were added and the resulting solution stirred at 70°C for 15 h. The solvent was evaporated in *vacuo* to give crude material which was chromatographed (0-20% EtOAc / cyclohexane) to give **7a** as a white solid (11.76 g, 33.4 mmol, 76%). ^1^H NMR (DMSO-*d6*): δ 5.94 – 5.89 (m, 1H), 3.97 – 3.90 (m, 1H), 3.3.75 – 3.68 (m, 1H), 2.35 – 2.34 (m, 1H), 2.01 – 1.86 (m, 2H)1.81 – 1.70 (m, 1H), 1.59 – 1.52 (m, 2H).

***N*-*trans*-4-((4-methoxy-3-(3-methylmorpholino)-1-(tetrahydro-2*H*-pyran-2-yl)-1*H*-pyrazolo[3,4-*d*]pyrimidin-6-yl)amino)cyclohexyl)-2-methylpropane-1-sulfonamide 16a**

A mixture of **4c** (1.0 g, 1.83 mmol), 3-methylmorpholine (0.93 g, 9.17 mmol), potassium hexamethyldisilazide in toluene (7.33 ml, 3.67 mmol), RuPhos (0.086 g, 0.183 mmol) and tris(dibenzylideneacetone)dipalladium(0) (0.084 g, 0.092 mmol), in 1,4-dioxane (29 mL) was purged with argon and heated at 110°C for 2 h. Further 3-methylmorpholine (0.93 g, 9.17 mmol), potassium hexamethyldisilazide in toluene (3.67 mL, 1.83 mmol), RuPhos (0.043 g, 0.092 mmol) and tris(dibenzylideneacetone)dipalladium(0) (0.042 g, 0.046 mmol) were added and the resulting suspension heated at 110°C for 1 h. The mixture was partitioned between ethyl acetate (60 mL) and water (100 mL), the aqueous phase extracted with ethyl acetate (2x 60 mL) and the organic layers combined, dried over Na_2_SO_4_, filtered and concentrated *in vacuo*. Crude material was chromatographed (8-70% ethyl acetate / cyclohexane) to give **16a** (190 mg, 0.34 mmol, 18%). ^1^H NMR (DMSO-*d6*): δ 7.20 – 7.06 (m, 1H), 7.03 – 6.99 (m, 1H), 5.55 – 5.43 (m, 1H), 3.97 – 3.87 (m, 4H), 3.82 – 3.47 (m, 5H), 3.28 – 3.15 (m, 3H), 3.08 – 3.00 (m, 1H), 1.89 (d, *J* = 6.4 Hz, 2H), 2.10 – 2.03 (m, 1H), 1.96 – 1.86 (m, 4H), 1.78 – 1.41 (m, 5H), 1.36 – 1.28 (m, 4H), 1.10 – 0.98 (m, 10H); m/z 566.1 [M + H]^+^

***N*-(*trans*-4-((4-methoxy-3-(3-methylmorpholino)-1*H*-pyrazolo[3,4-*d*]pyrimidin-6-yl)amino)cyclohexyl)-2-methylpropane-1-sulfonamide 1**

To a solution of **16a** (0.180 g, 0.318 mmol) in MeOH (3.2 mL), acetyl chloride (0.023 mL, 0.32 mmol) was added and the resulting solution stirred at room temperature for 1 h. Further acetyl chloride(0.023 mL, 0.32 mmol) was added and stirred for a further 30 min. Solvent was evaporated *in vacuo*, and the mixture partitioned between sat. NaHCO_3_ (50 mL) and EtOAc (30 mL). The aqueous layer was washed with further EtOAc (30 mL) and the combined organics dried over Na_2_SO_4_, filtered and the solvent removed *in vacuo*. Crude material was chromatographed (0 – 50% EtOAc / cyclohexane) to give **1** (129 mg, 0.27 mmol, 84%). ^1^H NMR matches previously described batch. m/z 482.2 [M + H]^+^.

**3,3,3-Trifluoro-*N*-(*trans*-4-((4-methoxy-3-(3-methylmorpholino)-1*H*-pyrazolo[3,4-*d*]pyrimidin-6-yl)amino)cyclohexyl)propane-1-sulfonamide 19**

**19** was synthesised according to the procedure used for **1** (THP as protecting group): (72 mg, 0.14 mmol). ^1^H NMR (DMSO-*d6*): δ 11.97 (s, 1H), 7.36 – 7.32 (m, 1H), 6.97 – 6.90 (m, 1H), 3.99 – 3.89 (m, 4H), 3.81 – 3.70 (m, 2H), 3.65 – 3.51 (m, 3H), 3.27 – 3.19 (m, 3H), 3.17 – 3.10 (m, 3H), 2.70 – 2.58 (m, 2H), 1.95 – 1.86 (m, 4H), 1.39 – 1.22 (m, 5H), 1.02 (d, *J* = 6.6 Hz, 3H); ^13^C NMR (DMSO-*d6*): δ 162.93, 160.45, 160.08, 150.87, 127.87 (q, *J* = 130 Hz), 87.59, 70.97, 66.58, 53.78, 52.00, 51.11, 48.95, 45.49, 44.35, 33.18, 31.29, 28.75 (q, *J* = 30 Hz), 12.86; m/z 522.2 [M + H]^+^.


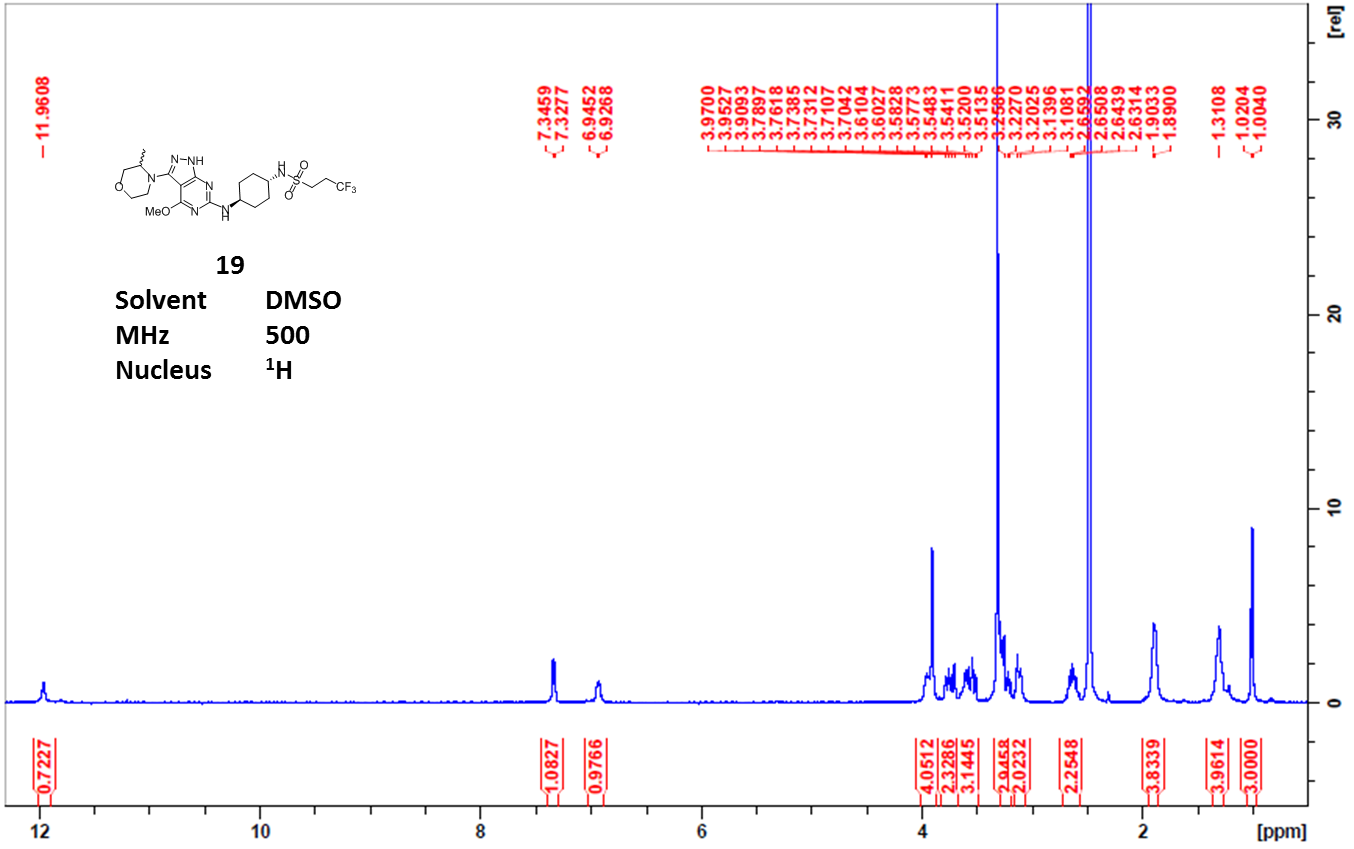


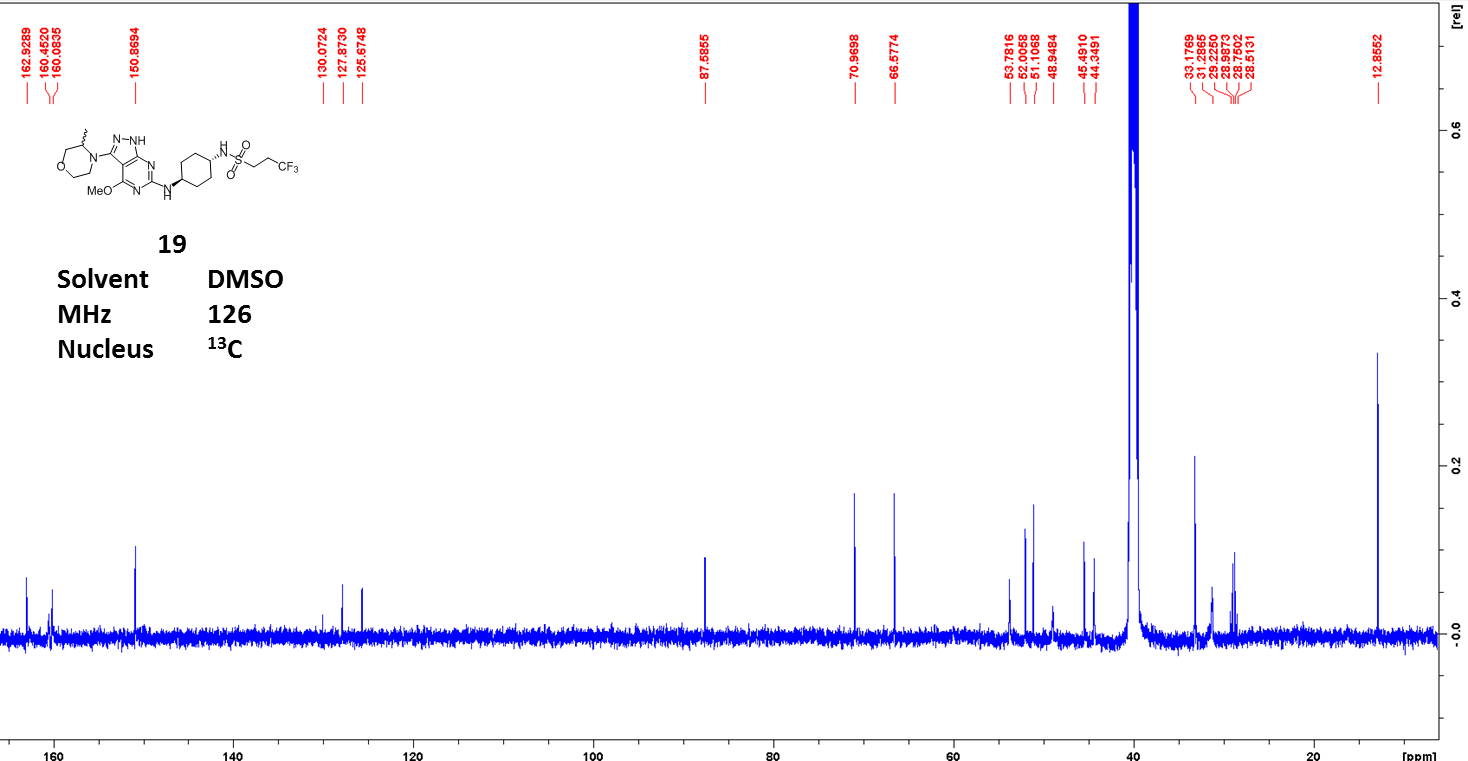


***N*-(*trans*-4-((4-methoxy-3-((*R*)-3-methylmorpholino)-1*H*-pyrazolo[3,4-*d*]pyrimidin-6-yl)amino)cyclohexyl)propane-2-sulfonamide (*R*)-20**

**(*R*)-20** was synthesised according to the procedure used for **1** (SEM as protecting group): (1.38 g, 2.95 mmol). ^1^H NMR (DMSO-*d6*): δ 11.97 (s, 1H), 6.98 – 6.86 (m, 2H), 4.00 – 3.91 (m, 4H), 3.82 – 3.71 (m, 2H), 3.67 – 3.53 (m, 3H), 3.29 – 3.21 (m, 1H), 3.19 – 3.11 (m, 2H), 3.08 – 3.01 (m, 1H), 1.95 – 1.88 (m, 4H), 1.40 – 1.29 (m, 4H), 1.22 (d, *J* = 6.8 Hz, 6H), 1.03 (d, *J* = 6.6 Hz, 3H); ^13^C NMR (DMSO-*d6*): δ 162.93, 160.46, 160.09, 150.87, 87.58, 70.97, 66.58, 53.78, 52.49, 52.22, 51.11, 49.01, 44.34, 33.49, 31.42, 16.92, 12.85; m/z 468.2 [M + H]^+^.


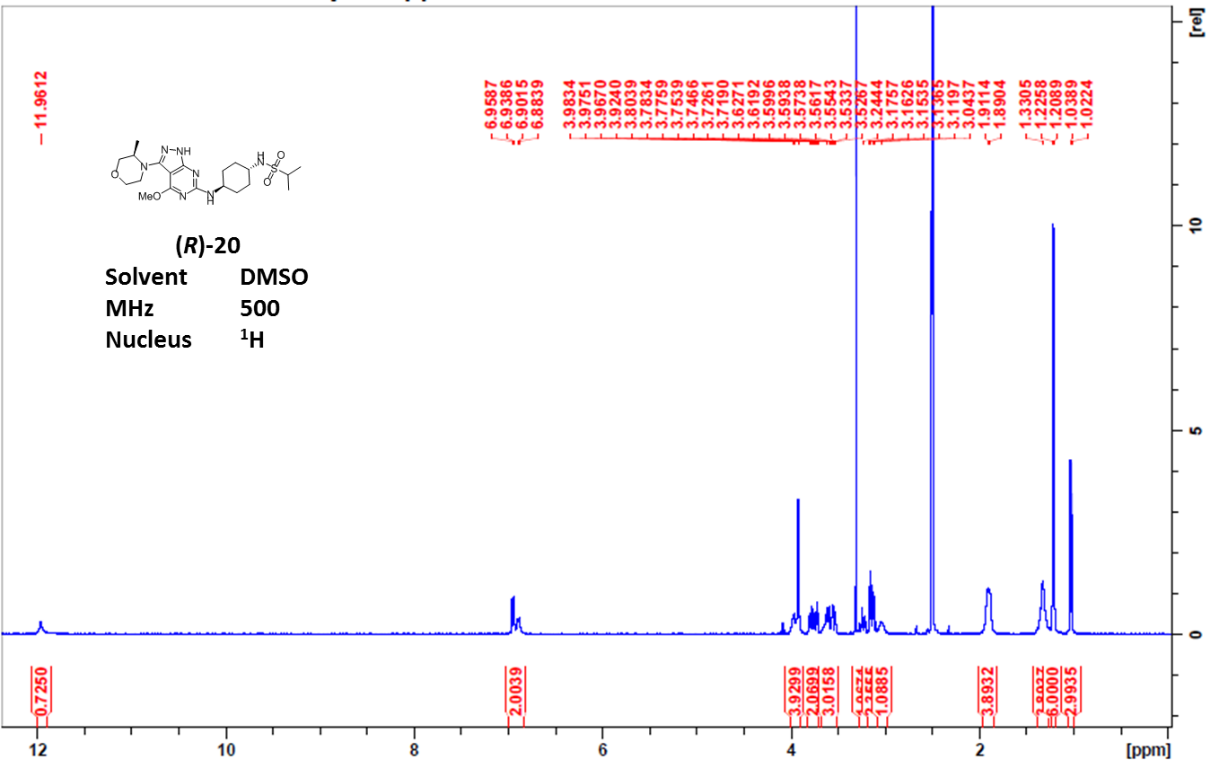


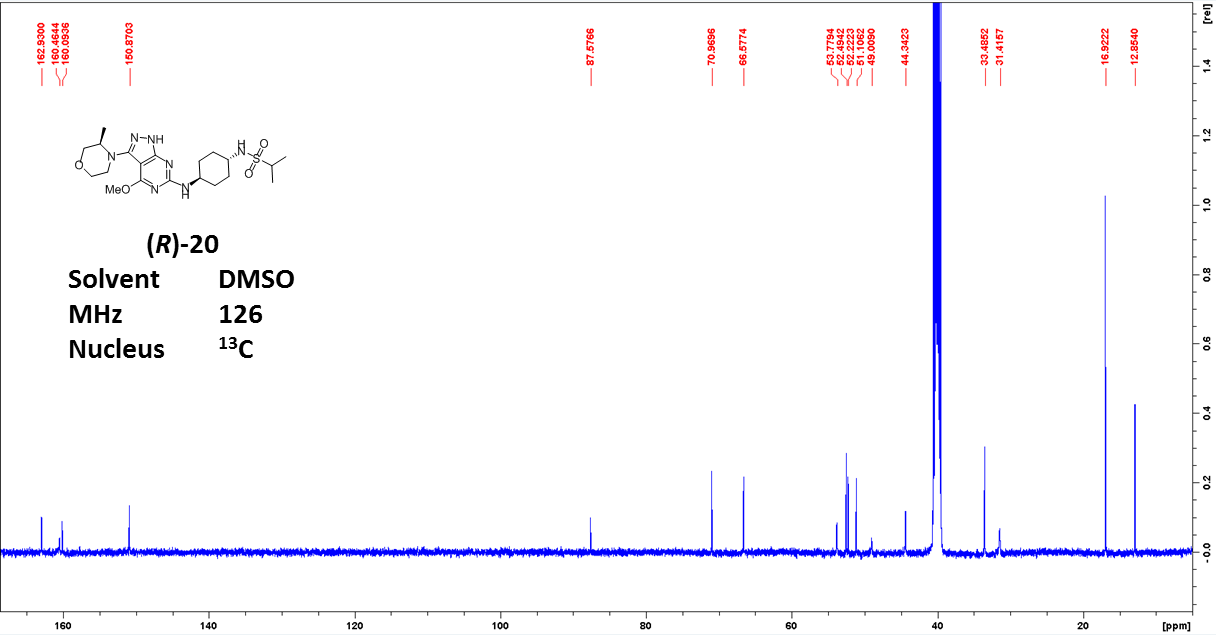


***N*-(*trans*-4-((4-methoxy-3-((*R*)-3-methylmorpholino)-1*H*-pyrazolo[3,4-*d*]pyrimidin-6-yl)amino)cyclohexyl)propane-2-sulfonamide (*S*)-20**

**(*S*)-20** was synthesised according to the procedure used for **1** (SEM as protecting group): (1.41 g, 3.0 mmol). ^1^H NMR (DMSO-*d6*): δ 11.96 (s, 1H), 6.96 – 6.86 (m, 2H), 3.98 – 3.89 (m, 4H), 3.80 – 3.69 (m, 2H), 3.64 – 3.51 (m, 3H), 3.27 – 3.19 (m, 1H), 3.16 – 3.08 (m, 2H), 3.06 – 2.98 (m, 1H), 1.94 – 1.83 (m, 4H), 1.38 – 1.26 (m, 4H), 1.21 (d, *J* = 6.8 Hz, 6H), 1.00 (d, *J* = 6.6 Hz, 3H); ^13^C NMR (DMSO-*d6*): δ 162.93, 160.46, 160.09, 150.87, 87.58, 70.97, 66.58, 53.78, 52.49, 52.22, 51.10, 49.01, 44.34, 33.48, 31.43, 16.92, 12.85; m/z 468.3 [M + H]^+^.


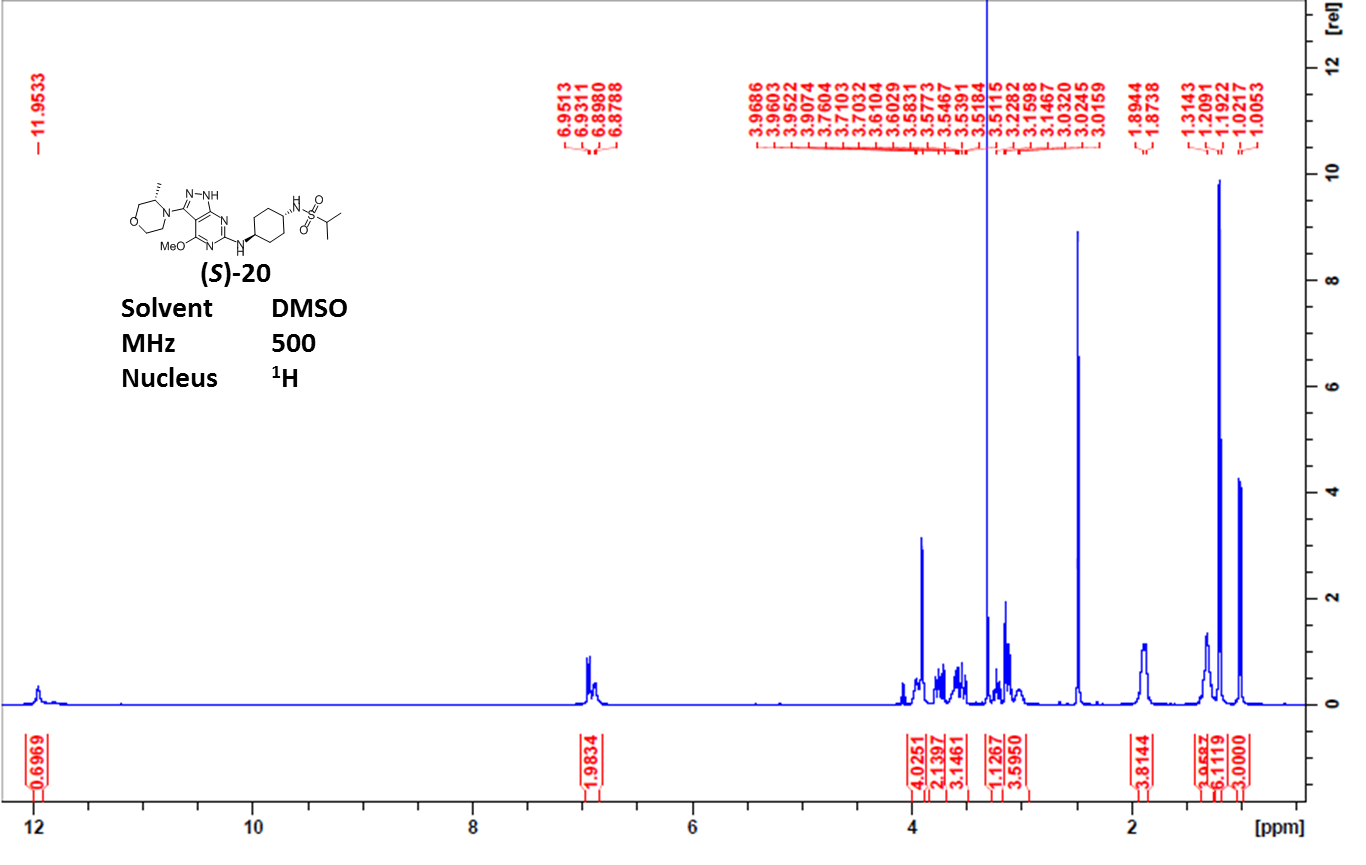


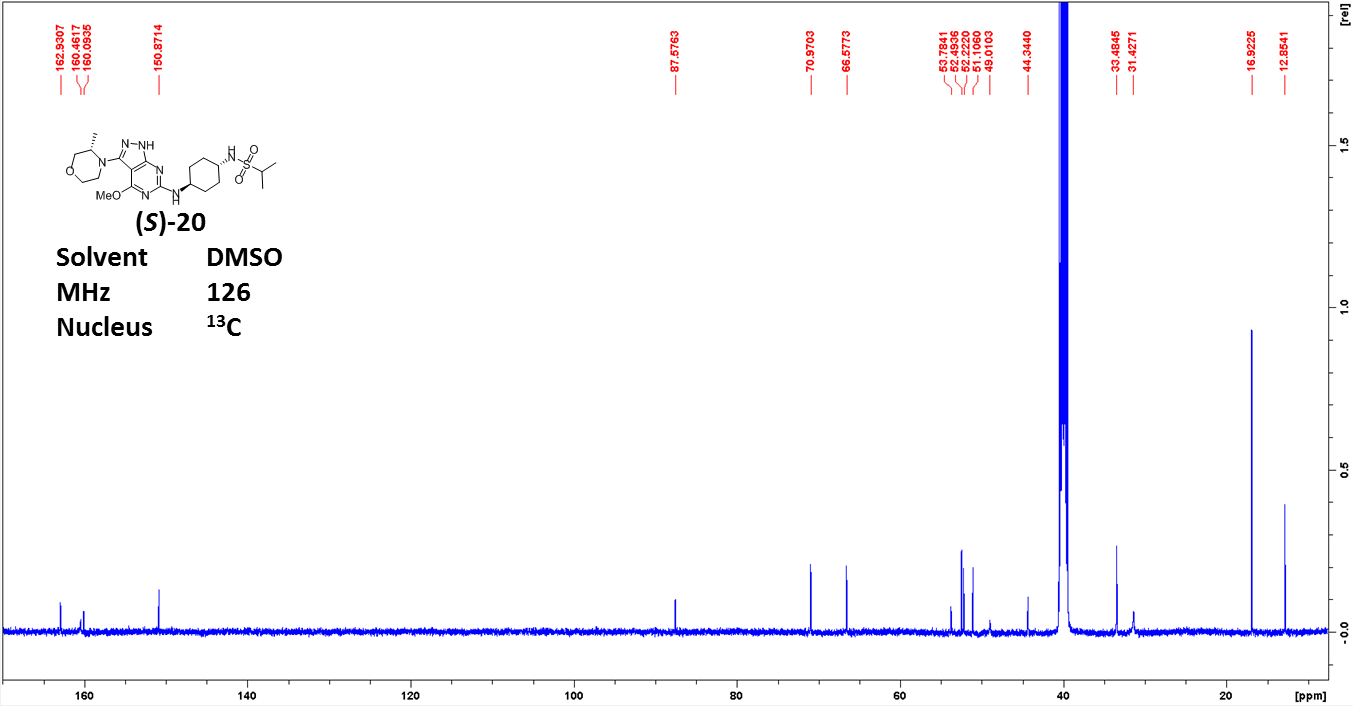


***N*-(*trans*-4-((4-methoxy-3-((*S*)-3-methylmorpholino)-1*H*-pyrazolo[3,4-*d*]pyrimidin-6-yl)amino)cyclohexyl)-*N*,2-dimethylpropane-1-sulfonamide 21**

**21** was synthesised according to the procedure used for **1** (THP as protecting group): (120 mg, 0.24 mmol). ^1^H NMR (DMSO-*d6*): δ 11.98 (s, 1H), 6.96 – 6.91 (m, 1H), 3.99 – 3.88 (m, 4H), 3.82 – 3.50 (m, 6H), 3.27 – 3.20 (m, 1H), 3.18 – 3.10 (m, 1H), 2.90 (d, *J* = 6.5 Hz, 2H), 2.69 (s, 3H), 2.11 – 1.92 (m, 3H), 1.68 – 1.57 (m, 4H), 1.44 – 1.32 (m, 2H), 1.03 – 0.99 (m, 9H); ^13^C NMR (DMSO-*d6*): δ 162.94, 160.45, 160.06, 150.88, 87.62, 70.97, 66.58, 57.83, 55.54, 53.81, 51.11, 48.85, 44.37, 31.62, 29.30, 28.70, 24.90, 22.70, 12.86; m/z 496.0 [M + H]^+^.


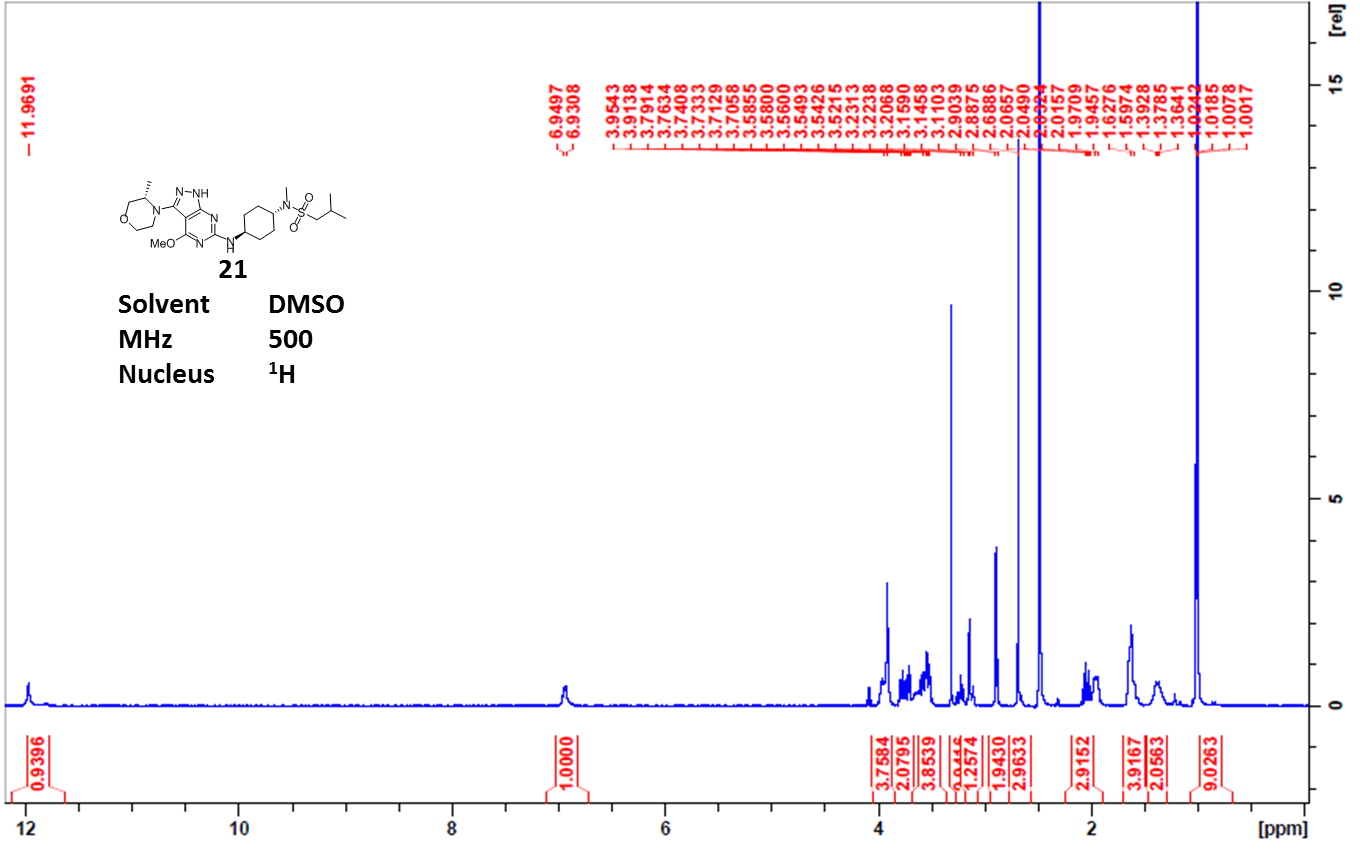


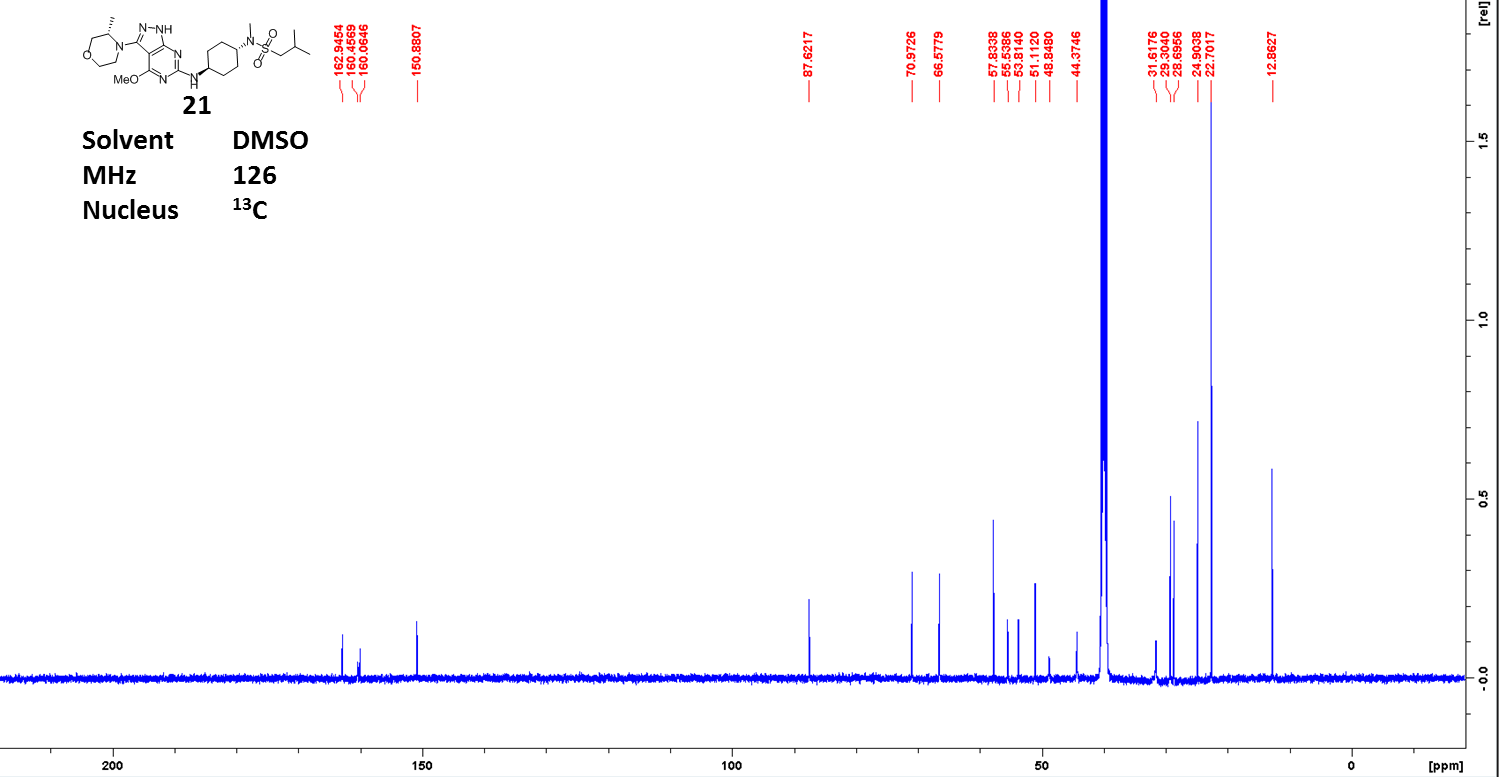


***N*-(*trans*-4-((4-methoxy-3-(3-methylmorpholino)-1*H*-pyrazolo[3,4-*d*]pyrimidin-6-yl)amino)cyclohexyl)-*N*-methyl-1-(tetrahydrofuran-2-yl)methanesulfonamide 22**

**22** was synthesised according to the procedure used for **1** (THP as protecting group): (46 mg, 0.09 mmol). ^1^H NMR (DMSO-*d6*): δ 11.97 (s, 1H), 7.02 – 6.96 (m, 1H), 6.94 – 6.89 (m, 1H), 4.15 – 4.08 (m, 1H), 4.00 – 3.88 (m, 3H), 3.81 – 3.70 (m, 3H), 3.64 – 3.51 (m, 3H), 3.26 – 3.04 (m, 4H), 2.09 – 1.99 (m, 1H), 1.94 – 1.77 (m, 5H), 1.70 – 1.61 (m, 1H), 1.36 – 1.21 (m, 4H), 1.01 (d, *J* = 6.6 Hz, 2H); ^13^C NMR (DMSO-*d6*): δ 162.93, 160.47, 160.10, 150.87, 87.57, 74.00, 70.97, 67.52, 66.58, 57.75, 53.78, 51.99, 51.10, 44.34, 33.41, 33.12, 31.52, 31.36, 25.33, 12.85; m/z 510.0 [M + H]^+^.


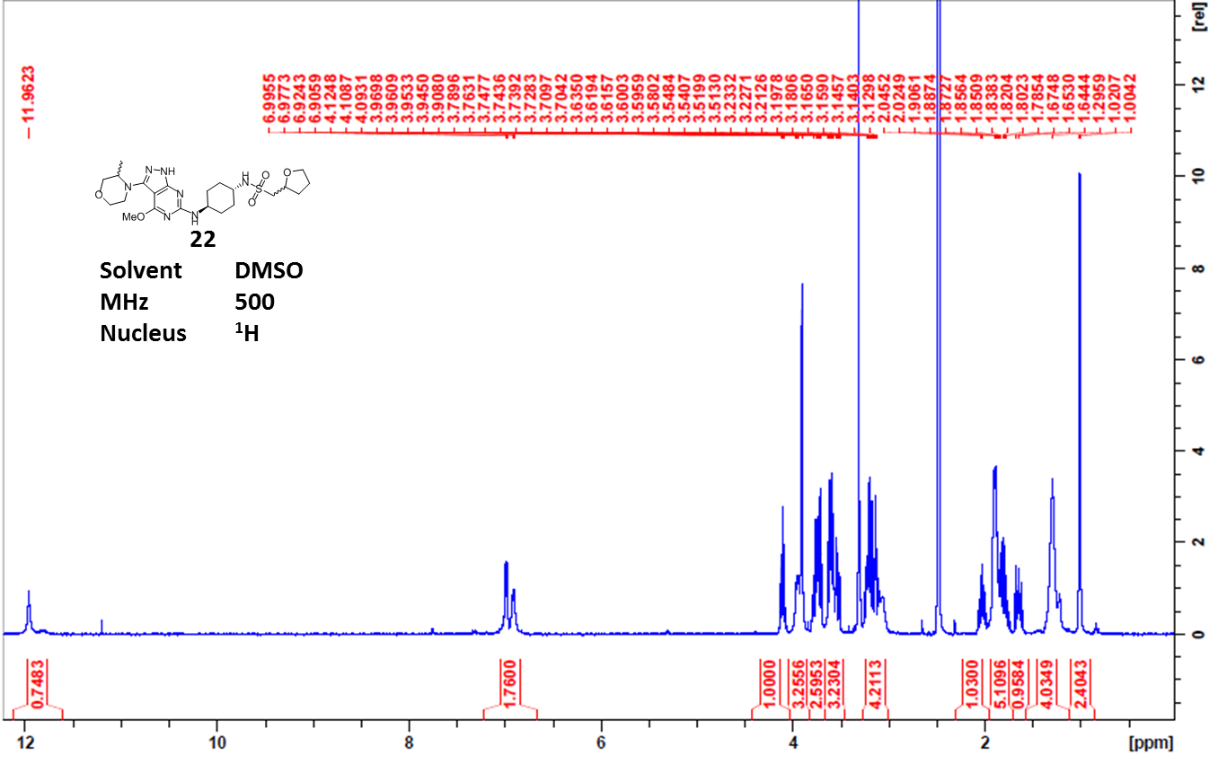


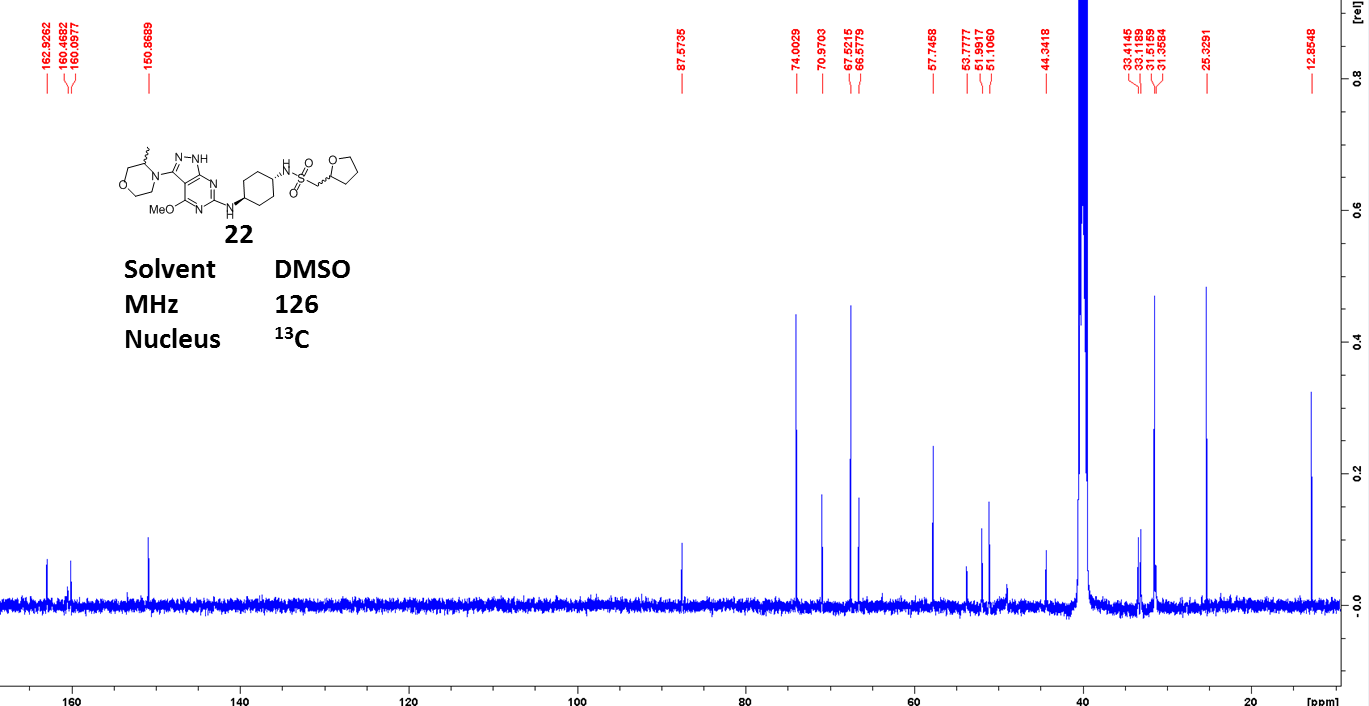


***N*-(*trans*-4-((4-methoxy-3-(3-methylmorpholino)-1*H*-pyrazolo[3,4-*d*]pyrimidin-6-yl)amino)cyclohexyl)-2,2-dimethylpropane-1-sulfonamide 23**

**23** was synthesised according to the procedure used for **1** (THP as protecting group): (30 mg, 0.06 mmol). ^1^H NMR (DMSO-*d6*): δ 11.96 (s, 1H), 6.97 – 6.90 (m, 2H), 4.00 – 3.88 (m, 4H), 3.81 – 3.69 (m, 2H), 3.63 – 3.50 (m, 3H), 3.26 – 3.02 (m, 3H), 2.94 (s, 2H), 1.94 – 1.86 (m, 4H), 1.36 – 1.23 (m, 4H), 1.07 (s, 9H), 1.01 (d, *J* = 6.6 Hz, 3H); ^13^C NMR (DMSO-*d6*): δ 162.93, 160.47, 160.10, 150.87, 87.58, 70.97, 66.58, 64.51, 53.79, 51.93, 51.11, 49.02, 44.35, 33.35, 31.55, 29.85, 12.86; m/z 496.4 [M + H]^+^.


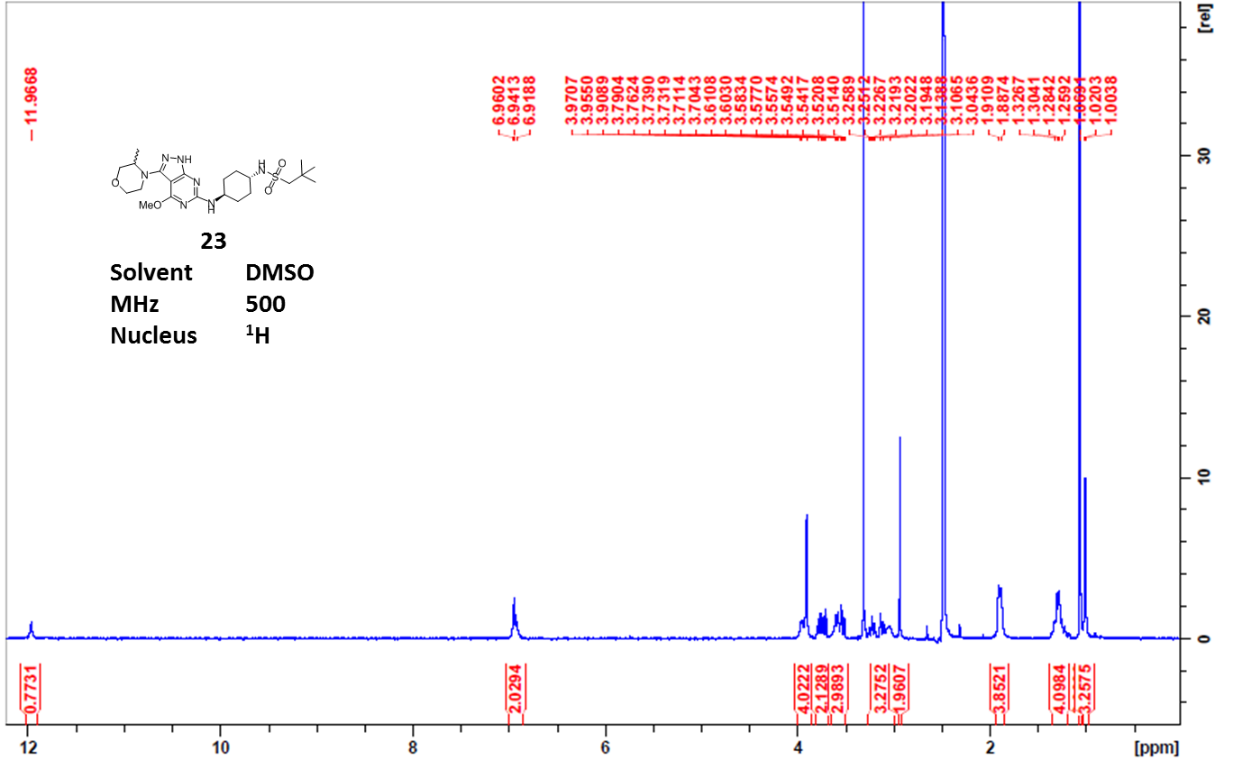


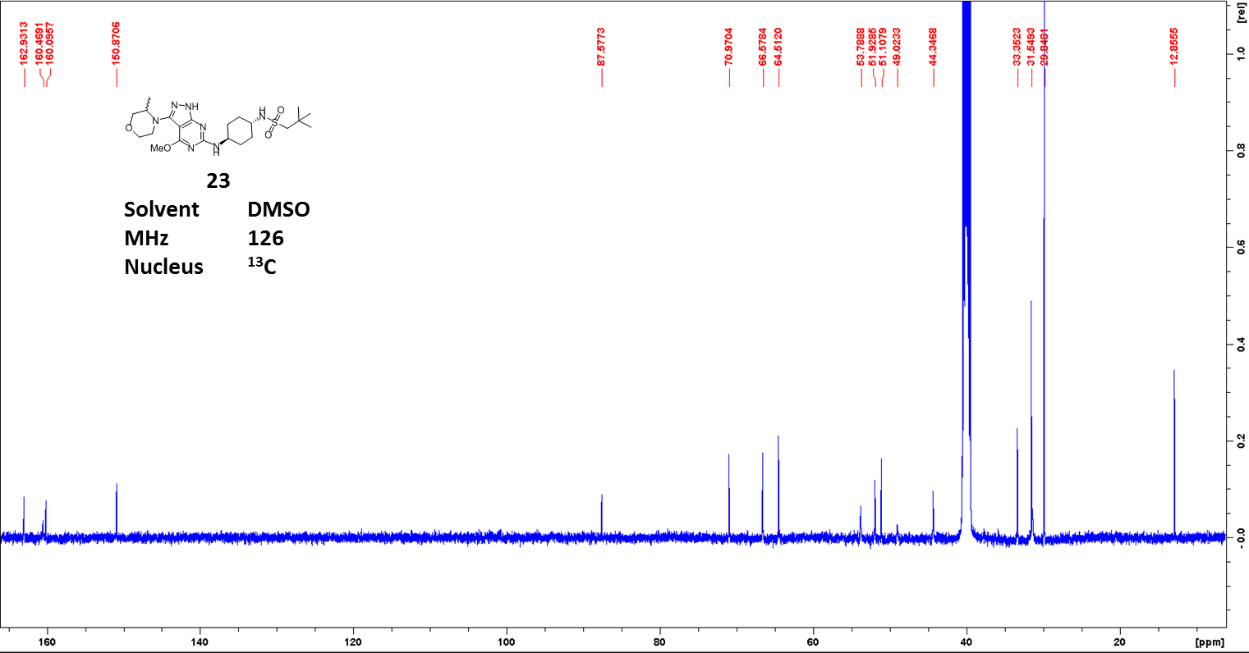

Supplement: Supplementary data 1 [file mmc1.docx]
